# Complete Genome Sequence of Anaeromyxobacter sp. Fw109-5, an Anaerobic, Metal-Reducing Bacterium Isolated from a Contaminated Subsurface Environment

**DOI:** 10.1128/genomeA.01449-14

**Published:** 2015-01-22

**Authors:** C. Hwang, A. Copeland, S. Lucas, A. Lapidus, K. Barry, T. Glavina del Rio, E. Dalin, H. Tice, S. Pitluck, D. Sims, T. Brettin, D. C. Bruce, J. C. Detter, C. S. Han, J. Schmutz, F. W. Larimer, M. L. Land, L. J. Hauser, N. Kyrpides, A. Lykidis, P. Richardson, A. Belieav, R. A. Sanford, F. E. Löeffler, M. W. Fields

**Affiliations:** a Center for Biofilm Engineering, Montana State University, Bozeman, Montana, USA; b DOE Joint Genome Institute, Walnut Creek, California, USA; c Biological Sciences Division, Pacific Northwest National Laboratory, Richland, Washington, USA; d Department of Geology, University of Illinois, Urbana-Champaign, Illinois, USA; e Department of Microbiology, Department Of Civil and Environmental Engineering, University of Tennessee, Knoxville, Tennessee, USA; f Biosciences Division, Oak Ridge National Laboratory, Oak Ridge, Tennessee, USA; g Department of Microbiology and Immunology, Montana State University, Bozeman, Montana, USA; h National Center for Genome Resources, Santa Fe, New Mexico, USA

## Abstract

We report the genome sequence of Anaeromyxobacter sp. Fw109-5, isolated from nitrate- and uranium-contaminated subsurface sediment of the Oak Ridge Integrated Field-Scale Subsurface Research Challenge (IFC) site, Oak Ridge Reservation, TN. The bacterium’s genome sequence will elucidate its physiological potential in subsurface sediments undergoing *in situ* uranium bioremediation and natural attenuation.

## GENOME ANNOUNCEMENT

The Oak Ridge Integrated Field-Scale Subsurface Research Challenge (IFC) site encompasses a U(VI)-contaminated area for conducting *in situ* bioremediation field research. Bioremediation efforts at the Area 3 site involved treatment of contaminated groundwater to optimize subsurface conditions for U(VI) reduction by indigenous microorganisms upon ethanol biostimulation (1). Concurrent cultivation efforts led to isolation of the anaerobic bacterium, Anaeromyxobacter sp. Fw109-5, from a ferric iron enrichment of subsurface sediments collected from monitoring well Fw109 located outside the treatment zone. While Anaeromyxobacter sp. Fw109-5 was isolated from Area 3, further evolutionary distance analysis indicated that sequences detected in the ethanol biostimulation treatment zone belonged to another distinct cluster of Anaeromyxobacter populations, whereas Anaeromyxobacter sp. Fw109-5 was more closely related to a cluster of Anaeromyxobacter sequences detected at another IFC treatment area that used alternative substrates for biostimulation (2). These results demonstrated that a diverse Anaeromyxobacter population exists at the IFC site and comparative genome analysis provides relevant information about their physiological capacity, which is crucial for future bioremediation designs.

The genome sequence for Anaeromyxobacter sp. Fw109-5 was determined with the Sanger sequencing method by the US DOE Joint Genome Institute (JGI). Genes were identified at Oak Ridge National Laboratory using the genome annotation pipeline based on the Prodigal gene prediction algorithm (3), followed by a round of manual curation using JGI’s GenePRIMP pipeline (4). Additional gene prediction analysis and functional annotation were performed within the Integrated Microbial Genomes (IMG) platform (https://img.jgi.doe.gov/cgi-bin/w/main.cgi) (5). Completed microbial genomes by JGI have been curated to close all gaps with greater than 98% coverage of at least two independent clones. Each base pair has a minimum quality value of 30 with a total error rate of less than 1/50,000. The genome sequence of Anaeromyxobacter sp. Fw109-5 is approximately 5.28 Mb in size, with a G+C content of 73.5%, and contains 4,549 putative genes, two ribosomal RNA operons (16S-23S-5S), 49 tRNA genes, and 4 other RNA genes. Of the 4,549 putative genes identified, 4,490 were protein-coding genes with functions predicted for 3,157 genes.

Other Anaeromyxobacter isolates used chlorinated phenols, oxygen, nitrate, nitrite, nitrous oxide, ferric iron, arsenate, manganese dioxide, U(VI), and Tc(VII) as electron acceptors (6–11). Unlike Anaeromyxobacter dehalogenans 2CP-C (GenBank accession number CP000251.1), Anaeromyxobacter Fw109-5 lacks putative reductive dehalogenase genes. Like its relatives, Anaeromyxobacter Fw109-5 lacks *nir* genes, but it has other genes associated with the denitrification pathway (12). Genome comparison of Anaeromyxobacter sp. Fw109-5 to other known metal reducers as well as related Anaeromyxobacter isolates will reveal unique traits and lead to an understanding of the contributions of these organisms for bioremediation.

### Nucleotide sequence accession numbers.

Anaeromyxobacter sp. Fw109-5 was assigned with the GenBank identification number CP000769.1 and NCBI reference sequence NC_009675.1.
